# Collectanea Medica: Consisting of Anecdotes, Facts, Extracts, Illustrations, &c.

**Published:** 1826-04

**Authors:** 


### COLLECTANEA MEDICA:

CONSISTING OF

### ANECDOTES, FACTS, EXTRACTS, ILLUSTRATIONS, &c.

*Relating to the History or the Art of Medicine, and the Collateral Sciences.*

Floriferus, ut apes, in saltibus omnia libant,

Omnia nos, itidem, depascimur aurea dicta.

---

**Art. I.**—*On the Mixture and Adulteration of Wines.* [From the "*History of Ancient and Modern Wines.*" London, 1824.]

Although there is no positive standard of taste in wines, and the demand for particular sorts is liable to great fluctuation, from the influence of fashion and caprice; yet there are certain qualities which may be deemed essential, and with respect to which all men are agreed. Some persons may like the sweet, others the dry wines; some the light, others the stronger kinds; but no one will choose a flat and insipid vintage, in preference to that which is distinguished by the fulness of its body, and richness of its flavour and perfume; or, when purchasing a supply, will be disposed to overlook the equally indispensable requisites of firmness and durability. These qualities, however, are seldom found combined, except in first-rate wines, which, being grown in very limited quantity, sell at high prices, and are procured with difficulty. Indeed, generally speaking, they may be said to be little known; for many of them, being the produce of private vineyards, are never to be purchased, or, if they are, must be bespoke before the vintage; and, of the remainder, a considerable portion is reserved by the farmer or merchant, for the purpose of mingling with those of inferior quality. Even the secondary wines are not easily to be had pure and unadulterated, but frequently undergo preparation before they are brought into commerce. Of some of these preparations, and of the manner in which they affect the qualities of the commodity, mention has been made in the foregoing chapters. I shall now proceed to inquire more particularly, to what extent they are practised in different countries,—how far they are rendered necessary by the operation of natural causes,—and in what degree they are reconcileable to the rules of fair and honourable dealing.

The number of hands through which wine usually passes before it reaches the consumer,—the great difference of price between the first-rate and the inferior sorts,—and the prevailing ignorance with respect to their distinguishing characters, afford so many facilities and temptations to fraud and imposition in this branch of trade, that no buyer, however great his caution, however just his taste, is wholly secure against them. It is undoubtedly the interest of the grower to raise as large a quantity of the prime kinds as he possibly can; and it is equally the interest of the merchant to gain the favour of his customers, by furnishing them with wines which give satisfaction. But, let the growers be ever so honestly inclined, they cannot always supply the commodity in as equal and perfect a state as they could wish : for, not to mention those defects in the nature of the soils, which necessarily restrict the production of fine wines within certain limits, there is a continual variation in the qualities of every sort of wine, accordingly as the seasons prove more or less favourable. Good vintages are, in fact, of much rarer occurrence than is commonly imagined ; at least, in those countries where the grape does not attain an early maturity. For example, in the Bordelais, the wines of 1819 alone claim superiority among those of the last eight seasons; in the Alto Douro, about ten of the last fifty years have been distinguished by the general excellence of the produce: while, on the banks of the Rhine, the number of propitious years since the commencement of the eighteenth century, does not exceed fifteen. Of the intervening seasons, many have been so absolutely bad, as not to allow the grapes to ripen thoroughly, or to be gathered in such condition as to yield a wine fit for drinking. Under these circumstances, the farmer, or merchant, who has been so fortunate as to save any portion of the better vintages, uses it for the purpose of mingling with such feeble and perishable wines as would otherwise remain unsaleable, and could not be preserved for any length of time in their natural state. Even in good years, however, there are many wines that rank above the ordinary kinds, which will not bear long keeping, or distant carriage, without some sort of preparation. Thus the wines of Torins, in the Mâconnais, although by no means deficient in spirituosity or flavour, retain their quality a much shorter time, and improve less when pure, than when they are mixed with the growths of the neighbouring territorities of Chénas and Romanèche, which are of a stronger character, though they fetch only about the same price. On the other hand, it is sometimes desirable to mix the rougher and high-coloured red wines with a certain portion of generous white wine, which softens their harshness, and renders them much sooner drinkable than they would otherwise be; for which reason these mixtures are by many preferred to the purer kinds, which require to be long kept before their full flavour and aroma are developed.

In the Austrian dominions, and other parts of Germany, it was formerly a rule in the wine trade, that, along with every cask of good wine, the buyer should, at the same time, be obliged to take one of inferior quality. This regulation, though calculated to relieve the grower from much risk and trouble, and occasionally to enable individuals to secure a cask of choice quality, must, on the whole, have had an injurious influence on the character of German wines; as it, in a manner, compelled the dealers to equalize their stock, by mixing good and bad together, and to send them thus mingled into the market, as the best sorts which the country was capable of producing.

The same thing will happen whenever the demand for any particular class of wines greatly exceeds the supply. The general use of port wine in England, for instance, has occasioned an increased demand for it at Oporto. The London merchant, in sending his orders, generally requests his correspondents to furnish him with wines of superior quality: but, out of the sixty or seventy thousand pipes annually made in the Cima do Douro, there are, perhaps, scarcely five thousand to which that character will properly apply; and these are not to be found in any one store, but are divided, in different proportions, among the several merchant exporters. The whole number of pipes commissioned for London may amount to twenty-five thousand. Supposing, then, that five thousand pipes of first-rate wine, if they are to be had, should be sent in their genuine purity, it is evident that, to complete the order, twenty thousand of inferior quality must be added; and, as a large proportion of the latter would not bear sea-carriage without some preparation, the shipper is forced to mix them with brandy, which, though it may prevent them from spoiling, renders them, in other respects, worse than before, as it destroys what little flavour they originally possessed. If the two sorts of wine come into the market in the respective conditions just mentioned, the one will be eagerly purchased by all who can afford the price; while the other will find few or no buyers, and will probably remain a useless incumbrance in the cellars of the importer. Or, should the difference of value between the two kinds be so great as to deter the majority of his customers from purchasing the first-rate wine, while the profit to be derived from the sale of the inferior is comparatively trifling, the importer will be induced to withhold the best from the market, and to employ it in raising the quality of the others to that standard which shall enable him to command the highest price for the whole. What the London merchant is here supposed to do, is actually performed by the merchant at Oporto. Unable to answer the call for the finest wines, and knowing, besides, that, if he were to send any considerable portion of them in a pure state, along with the more common kinds, of which the great bulk of the cargo must necessarily consist, he would run some risk of having the latter returned upon his hands; or, at all events, would in future experience much difficulty in disposing of such part of his stock: he accordingly resorts to the expedient of mingling the one with the other, so as to bring the whole to a nearly uniform degree of strength and flavour. The consumers of port, having their palates already habituated to this artificial compound, and being prevented by high duties from repairing to those countries which yield more delicate vintages at a cheaper rate, must rest contented with the manufactured liquor; and their only choice lies between the few samples which have accidentally or designedly been allowed to retain somewhat of their original excellence, and those which, from natural badness or adulteration, are nearly deprived of all pretensions to the character of wine. If it were not that an article sold as genuine really ought to be such, no great blame would attach to the Oporto merchant on this occasion. He only pursues the course which is most conducive to the advancement and permanence of his trade. Nor can those who think themselves obliged to imbibe daily a certain quantity of port wine reasonably complain, if he provides them with a regular and plentiful supply of their favourite beverage, of as good quality as the variations of the seasons, the extent of the orders received, and the other circumstances formerly stated, enable him to send.

In those provinces of France which yield the choicest wines, and carry on the most extensive trade in this commodity, the manner of proceeding is somewhat different. There the first-rate growths being always in much request, and readily finding purchasers at the highest prices, are carefully preserved in their genuine purity. If they were mixed with the inferior sorts, the delicate flavours, for which they are chiefly prized, would be almost entirely destroyed; and the value of the compound would not compensate the sacrifice it required. The French merchants, therefore, keeping their finest wines pure, use only the secondary kinds, especially those which possess much spirit and body, for mixing with such as are of too thin and feeble quality to answer the purposes of commerce. In this way a considerable portion of the ordinary wines of Medoc, and other parts of the Bordelais, are rendered fit for exportation, and come to us under the name of *claret;* bearing about the same relation to the prime growths of the Gironde that common port wine bears to the finest produce of the Alto Douro. As this species of compound is in general demand, and the district does not afford a sufficient quantity of the stronger kinds for the manufacture, the dealers (as I have already had occasion to mention,) are obliged to supply the deficiency by large importations from the more southerly provinces of France, and even to have recourse to the thick and heavy vintages of Spain, which, though deficient in flavour, possess the other requisite qualities. In making these mixtures, the object of the Bordeaux merchant is not to imitate the produce of the first-rate vineyards, which he certainly could not accomplish, though he were to attempt it,—but merely to correct the defects of the common wines of the country, and to enable him to export them in larger quantities than he could otherwise pretend to do. The indefinite term claret does not pledge him to furnish wine of any particular growth; and, if he only sells what goes by that name at a moderate price, the light wines of Medoc, strengthened and improved in flavour by an addition of Hermitage, may well satisfy his customers: but, if he vends the compounded liquor as the genuine produce of Latour or Château Margaux, and exacts the price of a first-rate vintage, he resorts to an artifice, which will not succeed with the skilful, and ought not to be practised against the inexperienced.

Although the same observations will, in general, apply to all attempts to counterfeit the choicer wines, yet there are certain kinds, to the distinguishing qualities of which a somewhat nearer approach may be made by artificial admixtures. Of this description are the luscious sweet wines, the flavours of which, though often very powerful, are less delicate than those of the red class; and are, besides, to a certain degree, obscured by the undecomposed saccharine matter in which these liquors abound. It is probably owing to the last-mentioned circumstance, or to the similarity of the grapes from which they are made, that many of them, though raised on different soils, resemble each other so closely, as to render their discrimination a matter of some nicety. Several of these analogies have been pointed out in the course of the preceding chapters; and they sometimes occur in cases where we should least expect to find them, as, for instance, in the *vin de paille*

of the Hermitage, which comes so near to white Constantia in all its qualities, that it might be readily mistaken for that celebrated growth. It is also worthy of remark, that, in proportion as the luscious wines advance in their age, their characteristic differences are less perceptible. Thus, Cyprus wine, which every one can recognise, when new, by its tarry taste, gradually loses this disagreeable quality, and becomes at last hardly distinguishable from the thick sweet wines of other countries; and the produce of the Frontignan vine, which, of all the muscadine kinds, possesses at first the most marked taste and perfume, has been found to acquire, by long keeping, the exact flavour of Malaga. These various coincidences probably suggested the idea of forming such imitations of the rarer and more costly varieties of dessert wines, as might serve as substitutes for them in those countries where the genuine kinds are seldom met with. We have seen that the Romans contrived, by means of artificial compounds, to supply the place of some of the most esteemed Greek wines, with which, until about the time of Julius Cæsar, they were but scantily provided. The increasing consumption of the capital held forth great encouragement to this species of manufacture, in all parts of the empire where wine was an article of commerce; and it is not a little remarkable, that the same territory which furnished the inhabitants of ancient Rome with an ample store of fictitious and adulterine liquors, should still carry on the same trade to the greatest extent. The abundance and variety of muscadine and other rich growths, obtained from the vineyards of Languedoc, give peculiar facilities for the preparation of these spurious wines, of which the dealers in the chief commercial towns of that province have industriously availed themselves. A considerable part of the *picardan*, and other inferior vintages, is appropriated to this purpose; and it is from the laboratories of Cette that great part of those specious compounds issue, which are consumed chiefly by the Germans, and other northern nations, under the names of Malaga, Alicant, Madeira, &c.

If we look into those receipt-books which are in every one's hands, we shall find that the rules given for the making and imitating of wines with other fruits than the juice of the grape, are chiefly applicable to the sweet kinds. The excess of sugar used in the preparation of these liquors serves to cover the coarse and harsh flavours of the other ingredients; and the manufacturer generally finds it expedient to leave a large portion undecomposed. Were he to prolong the fermentation until a dry wine was produced, it would, in most instances, be found quite intolerable.

As the mixing of foreign wines affords great opportunity for frauds on the revenue, as well as for the introduction of a variety of deleterious compositions which are designed to pass for wine, but have nothing in common with it except the name, the practice has been strictly prohibited by legislative enactments. In the reign of Edward III. we find a law, directing that assay of all the wines imported should be made, at least twice a year, in every town; and that such as were found to be spoiled or corrupted should be cast out, and the vessels in which they were contained broken in pieces. It appears, indeed, somewhat doubtful whether the expressions "*purrez ou corrumpuz,*" in the original, have reference so much to the sophistication, as to the natural corruption of wines: but, that the vintners of that time were guilty of various malpractices, may be inferred from the terms of the preamble, which charges them with selling wines, "*auxiben purrez comme seyns,*" at arbitrary and exorbitant prices, to the great injury of the public. By the 12th Car. II. cap. 25, sect. 11, it is ordered, "That no merchant, vintner, wine-cooper, or other person, selling or retailing any wine, shall mingle or utter any Spanish wine mingled with any French wine, or Rhenish wine, cyder, perry, stummed wine, honey, sugar, syrups of sugar, molasses, or any other syrups whatsoever; nor put in any isinglass, brimstone, lime, raisins, juice of raisins, water, nor any other liquor nor ingredients, nor any clary, or other herbs, nor any sort of flesh whatsoever;" and that merchants or wine-coopers so mingling their wines shall forfeit one hundred pounds, and the vintners forty pounds, for every such offence. This statute, which may serve to show the heterogeneous nature of the ingredients commonly employed by fraudulent dealers, is however far too indiscriminate in its prohibitions; as it precludes the merchant and vintner not merely from using various materials for heightening and altering the flavours of their wines, but even from having recourse to such substances as are generally acknowledged to be necessary for giving them that brightness and purity, without which they will neither find ready purchasers, nor keep for any length of time. Though repeatedly called into operation as far as concerns the mixture of liquors, I am not aware that it ever has been enforced against any of the other processes; but that, upon the whole, it has had little effect in stopping the manufacture of fictitious wines, is a matter of just lamentation. The increased demand for particular kinds of foreign wine,—the difficulty of procuring the best growths in sufficient abundance,—the high duties successively imposed on the introduction of the genuine commodity, and various other circumstances affecting its supply and consumption,—have all tended to encourage the manufacture of spurious sorts in this country. That the quantity drunk under the names of Port and Madeira especially, far exceeds the quantity imported, has long been notorious. The account given by Addison of the practices of the wine-brewers in his time, which, though somewhat highly coloured, there is no reason to suppose exaggerated with respect to the more important particulars, proves how completely they set at nought the interdict above cited. "There is," says that writer in one of his periodical essays,* "a certain fraternity of chemical operators, who work underground in holes, caverns, and dark retirements, to conceal their mysteries from the eyes and observations of mankind. These subterraneous philosophers are daily employed in the transmutation of liquors, and, by the power of magical drugs and incantations, raising, under the streets of London, the choicest products of the hills and valleys of France. They can squeeze Bordeaux out of the sloe, and draw Champagne from an apple. Virgil, in that remarkable prophecy,—

---

* Tatler, No. 131.

*Incultisque rubens pendebit sentibus uva—*

'The ripening grape shall hang on every thorn,'—

seems to have hinted at this art, which can turn a plantation of northern hedges into a vineyard. These adepts are known among one another by the name of wine-brewers; and, I am afraid, do great injury, not only to her majesty's customs, but to the bodies of many of her good subjects."

The same manufacture still flourishes; but the reduction of duty on Cape wines enables the adepts of the present day to employ, as occasion may require, a more substantial and convenient menstruum for their preparations, than that formerly used. By mixing these wines with the lees of other kinds, and tinting and compounding them with various drugs, they endeavour to counterfeit the more costly vintages of Spain and Portugal, and even of France: but, though they may sometimes succeed in imposing on the unwary, the predominant flavour of the produce of the Cape, which, with all their art, they can seldom conceal, generally betrays the nature of the mixtures, and renders them as unpalatable as they are unwholesome and worthless. The high impost on the choicer wines, however, holds forth so strong a temptation to embark in this disreputable trade, that we must lay our account with its continuance, until the return to more moderate and equal rates shall remove the causes from which it has chiefly sprung.

It has been observed, that, by the statute of Charles II., several substances are forbidden to be mixed with wine, which in themselves are not only innoxious, but deemed conducive to its purity and right preservation. In this respect our laws are not singular. The ordinances of the Imperial Diets are conceived in the same spirit; denouncing the use of lime, gypsum, sulphur, and even milk, and passing unnoticed the more censurable adulteration with lead: in conformity, as it would seem, to the prejudices of the ancients, who, not suspecting the dangers of the practice, often boiled their wines in leaden vessels, while they regarded the admixture of other mineral substances as highly injurious to health. At what period, or in what country, the deadly practice of adding litharge to acescent wines was introduced, is uncertain. Beckmann inclines to the opinion that it originated, or at least was first detected, in France; the earliest mention of that species of sophistication being found in an ordinance of the French police, which bears the date of 1696. That it had been known for some time previously, may, however, be inferred from the fact that, in the same year, several individuals in the duchy of Wirtemberg were poisoned by drinking wine sweetened with ceruse, of the employment of which the dealers made no secret; appealing to the authority of certain learned physicians, who pronounced the practice to be harmless, and sanctioned it by their own example. This defence did not allay the alarm. An inquiry was ordered into all the circumstances of the case; and, by the advice of the court physicians, who assured the government that both litharge and sulphur, but especially the latter when combined with bismuth, were exceedingly unsafe ingredients, the use of these substances in the preparation of wine was thenceforth declared to be a capital crime. In the following year, some offenders against this law were punished by banishment or hard labour; and a wine-cooper at Eslingen, who afterwards ventured to revive this nefarious trade, and had induced several persons, in various places, to follow it, was condemned to lose his head, while the possessors of the adulterated wines were severely fined, and the wines themselves thrown out.

This mode of curing, or rather disguising, acidity in wines, is unfortunately not altogether unknown in this country; but there is reason to believe that it is seldom or never resorted to, since the dealers have become apprised of its dangerous consequences. There is, indeed, little occasion for such a remedy in the case of the full and strong vintages, with which we are so abundantly supplied from Spain, Portugal, and Madeira. If employed at all, it can only be for the purpose of correcting the harshness incident to some of the lighter white wines, such as those of the Rhine, Moselle, or the Cape, and the inferior kinds of Teneriffe. When these wines have an unusual degree of sweetness, a darker colour than their age and body seem to warrant, and particularly when the use of them is followed by pains of the stomach, we may presume that they have been adulterated with lead. The presence of that mineral may be easily detected by adding to such wines a solution of the hydrosulphuret of potash, or water impregnated with sulphuretted hydrogen gas, which gives a black precipitate. The prussiate of potash is occasionally employed for the same purpose; a drop or two being sufficient to show a white or greyish precipitate in any fluid in which lead is contained.

According to some authors, lead is not the only poisonous metal used in the preparation of wines. The Spaniards are charged with having had recourse to arsenic, and even to corrosive sublimate, in order to fine their vintages, and render them more firm and durable; and the Dutch, also, are said to have prepared in the same manner such French wines as they shipped for their colonies. If these accusations be not unfounded, we may well exclaim with Pliny, "How can wine possibly prove innoxious, when it is mixed with so many destructive ingredients?"

Such, however, is the influence of custom in reconciling the palate to certain tastes, that wines are sometimes rendered more saleable by having qualities imparted to them, which in themselves are absolutely repulsive. We are apt to question the refinement of the ancients, who could relish the adventitious bitterness given to the produce of their vines, by means of pitch, rosin, and salt water; and yet are pleased with the coarse acerbity and astringency which port wine occasionally receives from the addition of alum, sloes, or oak-bark. We disdain the combination of odoriferous herbs, with which they scented their inferior wines; while our own are not unfrequently perfumed by an admixture of nitrous ether.

As a high colour is generally, though sometimes erroneously, considered a criterion of the excellence of particular wines, and as the vintages of unfavourable seasons are almost always deficient in this particular, it is frequently supplied by artificial means. For this purpose a variety of colouring matters are employed, such as the elder-berry, wortle-berry, privet, beet-root, tournesol (*croton tinctorium*), logwood, Brazil-wood, &c.; all of which, though they may improve the tint, deteriorate the flavour and durability of the wine. It is well known that port wines used to be prepared in this manner; and, though the company of the Alto Douro may have succeeded in extirpating the elder-tree from the district, yet they left the pokeweed (*phytolacca decandra*), the fruit of which has been found to answer equally well. The colour imparted by such materials, however, is seldom a pure red, but approaches more to violet, unless when heightened with alum; and the fraud is apt to betray itself by the flat and herbaceous taste which the liquor acquires. According to Cadet, this species of adulteration may be always easily detected by pouring into the suspected wine a solution of sulphate of alumina, and precipitating the alum by potash. If the wine is pure, the precipitate will have a bottle-green colour, more or less dark, according to the natural hue of the wine. Thus, the wines of Roussillon and Languedoc exhibit a dark green; those of Burgundy a bright green; and the *vin de pays* a green approaching to grey. If, again, the colouring has been artificial, the following will be the results:—

| | | |
|---|---|---|
| Tournesol will give a precipitate of | | a bright violet colour. |
| Brazil-wood | — | a brownish red colour. |
| Elder-berries, or privet | — | a brownish violet colour. |
| Wortle-berries | — | the colour of dirty wine-lees. |
| Logwood | — | a lake-red colour. |

Vogel recommends the acetate of lead for the same purpose; having remarked that, of the substances used for colouring wines, none will form a greenish-grey precipitate, like what is obtained from the genuine kinds, by means of the acetate. But the simple test pointed out to me by my friend, Dr. Prout, is equally satisfactory, and may be applied to the white as well as to the red kinds. On adding ammonia to wines which had the appearance of being genuine, he observed that the precipitate was of an olive-green colour; showing the analogy between the colouring principle and the vegetable blues, most of which are rendered red by acids, and green by alkalies. This conjecture is in some measure confirmed by the recent discovery of M. Breton, professor of chemistry at Paris, with respect to the cause of that disorder in wines known by the name of *tournure*. Wine thus affected acquires a disagreeable taste and smell, loses its red colour, and assumes a dark violet hue; which changes are found to proceed from the presence of carbonate of potash, in consequence of the decomposition of the tartar contained in the liquor. To restore the natural colour and flavour, if the disease be not of long standing, it is only necessary to add a small quantity of tartaric acid, which, combining with the potash, forms cream of tartar, as is shown by the subsequent deposition of crystals. In genuine wines, the colouring matter seems to partake of the character of a lake, partly held in solution by the excess of acid present, and partly combined with the earthy phosphates; for, in the precipitate obtained from these wines by means of ammonia, it appears in union with the triple phosphate of magnesia. Even the white wines of Xerez, Madeira, and Teneriffe, exhibit this mixed precipitate; their colouring matter being probably derived from the red grapes which enter into their composition. In fictitious wines, on the other hand, such as those procured from the black currant, gooseberry, orange, &c. the last-mentioned salt was thrown down by ammonia, but more gradually, in less quantity, and without any admixture.

Though the various sophistications, by means of heterogeneous ingredients, are not all equally reprehensible, it is very certain that none of them is calculated to supply or improve the qualities of genuine wine. Even the communication of artificial flavours derived from fruits and aromatic herbs, which is the most innocent of any, is apt to infect the liquor with a medicated taste, which, to a delicate palate, is immediately perceptible. The only legitimate mode of bettering wines, is by the addition of such constituents of the grape as the deficiencies of particular vintages appear to indicate; and they ought to be employed as much as possible during the fermentation, or before the wine is completely formed. To certain kinds, intended for distant climates, the admixture of a small quantity of brandy may be allowable, but never to such an extent as to overcome the original flavour; otherwise we impair their excellence, and risk their partial decomposition. In general, however, it may be observed, that the necessity for all these expedients will diminish as the culture of the vine improves, and as more skilful methods are adopted in the treatment of its produce. Every one will grant that prevention is better than cure; but in wine-making, as in other arts, this maxim is too much neglected. I may add, that the anticipated improvement will be greatly accelerated by the removal of those restrictions on trade which prevent us from frequenting the best and cheapest markets for wine, and compel us to receive so many cargoes of indifferent quality; at the same time that they tend to promote the manufacture of spurious compounds, and to encourage all manner of adulteration.

